# A Comparison of Snoring Changes With a Supine‐Avoidance Alarm Device Compared to Constant Positive Airway Pressure Treatment in Patients With Supine‐Predominant OSA

**DOI:** 10.1111/jsr.70128

**Published:** 2025-06-25

**Authors:** Matthew M. Rahimi, Andrew Vakulin, David Stevens, Peter G. Catcheside

**Affiliations:** ^1^ Flinders Health and Medical Research Institute: Sleep Health, (Formerly Adelaide Institute for Sleep Health), College of Medicine and Public Health Flinders University Bedford Park South Australia Australia; ^2^ Woolcock Institute of Medical Research Macquarie Park New South Wales Australia

**Keywords:** CPAP, posture shift, sleep apnea, snoring, supine‐avoidance

## Abstract

This study aimed to quantify how much snoring occurs in patients with supine‐predominant OSA and the comparative effectiveness of supine‐avoidance therapy versus CPAP to reduce objective measures of snoring. Participants had a 1‐week in‐home sleep posture assessment and a in‐home PSG study before being randomised to either CPAP or supine‐avoidance therapy for 6–8 weeks, then switched treatments for another 6–8 weeks. Snoring and treatment outcomes were examined in a subgroup of patients with supine dependent snoring. Baseline measurements showed snoring frequency was 48.9 [95% CI 16.7 to 188.7], 26.3 [95% CI 17.0 to 106.3], 57.5 [95% CI 16.8 to 190.7] snores/h for the whole group, supine snorer and non‐supine snorers respectively. Supine sleep as a percentage of total sleep was almost completely abolished with supine‐avoidance treatment (Baseline = 30.7% [95% 15.4 to 46.4], supine‐avoidance = 0.2% [95% 0.0 to 15.7]). In the whole group, CPAP significantly reduced overall snoring frequency although residual snoring remained. However, no treatment effect was observed with supine‐avoidance therapy in reducing snoring frequency (frequency of snores ≥ 50 dBA; baseline = 48.9 [95% 16.7 to 188.7], supine‐avoidance = 36.8 [95% 6.3 to 233.7], CPAP = 4.2 [95% 2.1 to 29.5] snores/h). In the sub‐group of supine‐predominant snorers, supine‐avoidance therapy was associated with reduced snoring frequency (Baseline = 26.3 [95% 17.0 to 106.3], supine‐avoidance = 14.3 [95% 3.0 to 18.1], CPAP = 4.6 [95% 2.6 to 8.9] snores/h). Supine‐avoidance therapy may not necessarily lead to a systematic reduction of snoring frequency in patients with supine‐predominant OSA but appears effective in a subgroup of patients.

## 1 Introduction

Snoring and obstructive sleep apnea (OSA) are part of a continuum of obstructed breathing in sleep and underpin the majority of referrals to a sleep physician for a sleep study (Larsson et al. 2003). Snoring reflects Starling resistor‐like behaviour of the upper airway where dynamic partial airway collapse limits airflow irrespective of inspiratory driving pressures (Wellman et al. 2014). Consequently, a portion of often substantial inspiratory effort is converted into tissue vibration and snoring with reduced airflow (hypopnoea) instead of normal unimpeded airflow and ventilation. Although OSA can include apneas without snoring, and snoring can be sustained for long periods that fail to meet traditional hypopnea scoring criteria, most respiratory events in OSA reflect transient hypopneas (Ratnavadivel et al. 2009) likely to be directly associated with snoring. Thus, up to 95% of all OSA patients snore heavily most or every night (Viner et al. 1991) and snoring frequency and intensity are strongly associated with respiratory and arousal disturbances in sleep (Viner et al. 1991; Wilson et al. 1999).

Habitual loud snoring without OSA affects somewhere between 15% and 54% of all middle‐aged adults, potentially making it a more common problem than OSA (Young et al. 1993; Enright et al. 1996). In clinical settings, snoring is a clear sign of a collapsible airway, but is considered less of a clinical concern than frequent transient obstruction, desaturation and arousal events that contribute to an elevated apnea hypopnoea index (AHI) in OSA. Nevertheless, habitual loud snoring without necessarily a high AHI may have important negative effects on the snorer and bed partner's sleep quality and health.

Snoring can substantially disrupt patient and particularly bed‐partner sleep, contributing to insomnia, psychosocial problems, and reduced quality of life for both snorers and their partners. Snoring has been linked to marital problems (Jones and Swift 2000), occasional acts of violence including homicide (Pelausa and Tarshis 1989), and is one of the primary reasons patients seek sleep treatments from ear, nose and throat (ENT) specialists (Marshall et al. 2007; Mcardle et al. 2001). Beyond social consequences, snoring may also pose independent health risks. Vibration from loud snoring has been hypothesized to contribute to vascular injury in nearby tissues, including the carotid arteries, although its long‐term significance remains unclear (Amatoury et al. 2006; Curry et al. 2002; Puig et al. 2005; Lee et al. 2008).

Upper airway collapsibility, and thus snoring, during sleep is strongly body position dependent and both are typically higher during supine compared to non‐supine sleep. Although the prevalence of supine‐predominant snoring is not well‐established, approximately 50% of all patients diagnosed with OSA show strong supine‐predominance of OSA severity, with an at least a 2‐fold higher supine versus non‐supine AHI (Adams et al. 2017). The majority of snorers without OSA also snore less in lateral compared to supine sleep (Loord and Hultcrantz 2007; Miyamoto et al. 1997). Supine‐avoidance devices can significantly reduce time spent sleeping supine and may be suitable for the one‐third of OSA patients with supine‐predominant OSA (Oksenberg and Gadoth 2019; Berry et al. 2019), as well as many snorers without OSA. However, their effectiveness in reducing snoring specifically remains to be demonstrated.

Despite the effectiveness of continuous positive airway pressure (CPAP) for reducing AHI, patient acceptance and adherence to treatment remains problematic (Rotenberg et al. 2016) and there remains a remarkable lack of objective data regarding effects on snoring, one of the key reasons for which patients may seek treatment. Supine‐avoidance via a simple non‐discomfort vibration‐based alarm device is effective in reducing sleepiness and has shown substantially greater treatment adherence compared to CPAP (Rahimi, Vakulin, Mcevoy, et al. 2024). Given a lack of data concerning OSA treatment effects on snoring, the primary purpose of this study was to compare snoring sound measured by snoring frequency, intensity and snoring‐related complaint reductions between supine‐avoidance therapy compared to CPAP. A secondary aim was to examine the prevalence of supine‐predominant snoring in patients selected on the basis of supine‐predominant OSA, and to test for potential differences in snoring and treatment adherence outcomes in patients with versus without co‐existing supine‐predominant snoring.

## 2 Method

Participants were from a previously reported randomised‐controlled cross‐over trial (SUPA trial) (Rahimi, Vakulin, Mcevoy, et al. 2024) selected on the basis of polysomnography (PSG) confirmed supine‐predominant OSA defined on the basis of a total apnea‐hypopnoea index (AHI) > 10 events per hour and a supine AHI at least twice that of non‐supine AHI measured from at least 4 h of recording with at least 30 min in supine and non‐supine postures. All available data from the full SUPA cohort were examined including a subgroup of patients who were also classified as supine‐predominant snorers based on a snoring frequency of at least twice that in supine compared to non‐supine positions. A total of 56 patients completed the SUPA trial and were available for this analysis. Following screening and consent, each participant completed a 1‐week in‐home assessment of sleep posture (inactive supine‐alarm device) and a full in‐home PSG study at baseline prior to randomisation to receive either CPAP or supine‐avoidance therapy as their initial treatment. Patients then remained on each allocated treatment for a total of 6–8 weeks before crossing over to the other treatment for a further 6–8 weeks, with a full in‐home PSG on allocated treatment repeated within the last week of each treatment arm of the study.

Baseline and follow up questionnaires including Snoring Severity Scale (SSS) (Lim and Curry 1999), assessment of quality of life (AQoL‐8D) (Richardson et al. 2014) and Epworth Sleepiness Score (ESS) (Johns 1992) were used to assess the effects of snoring on quality of life and sleep quality. There were also no significant improvements in subjective questionnaire measures. This may be due to the short treatment duration, limited sample size, or individual variability in the perception of snoring and sleep quality. Further studies with larger samples and longer follow‐up may be needed to detect changes in subjective outcomes.

### 2.1 Supine‐Avoidance Treatment

As described in our previous paper (Rahimi, Vakulin, Mcevoy, et al. 2024), supine‐avoidance treatment was achieved using a simple battery‐operated supine‐alarm device (Buzzpod, Gorman ProMed Pty. Ltd). The device is worn on the chest and records body position at 1 Hz, from which the onset and duration of supine alarm events are derived. A strong vibratory stimulus can be set to remain inactive or activate after five consecutive seconds of supine posture. Once triggered, the alarm stops upon a change to non‐supine but otherwise stays on for 2 min, then pulses every 5 s for 3 more minutes before timing out to conserve battery power. It supports up to 4 weeks of use before data download via USB.

### 2.2 Sleep and Snoring Assessments

PSG studies (Embletta MPR, Pleasanton, California, United States) included all standard recording channels according to the AASM rules (Berry et al. 2012).

All PSG signal data were exported to European data format (EDF) for sleep scoring and analysis, including snoring assessment using Compumedics software (Profusion4, Compumedics, Melbourne Australia). All sleep studies were scored by an experienced sleep technician blinded to the study treatment allocation. The primary study outcome was based on discrete snore events detected algorithmically (Profusion4, Compumedics) and categorised as soft (≥ 50 to < 60dBA), medium (≥ 60 to < 70 dBA) or loud (≥ 70 dBA) (Bignold et al. 2011) from which snoring frequency per hour of sleep was calculated based on sleep time in each stage and posture for each category of snoring and overall for all events ≥ 50 dBA.

Treatment adherence was determined using device recorded usage time per night during the course of each treatment. Blood pressure was measured before bedtime on PSG nights using a standard automatic digital sphygmomanometer.

### 2.3 Statistical Analysis

Analysis was performed on all available data. Given the count‐based nature of the primary snoring frequency outcome, negative binomial regression analysis was used to test for effects of treatment condition (baseline, supine‐avoidance and CPAP), including treatment order, on snoring frequency and intensity (mild, moderate and severe) in‐line with recommendations for zero inflated count data (Yau et al. 2003; Alexander 2012). All other comparisons between conditions in normally distributed continuous outcomes were examined using linear mixed effects model analysis using an auto‐regressive covariance structure and subjects as a random effect, each with their own intercept, to account for the repeated measures design and expected inter‐individual variability. IBM SPSS (Version 25) was used to perform all statistical analysis. All data are reported as mean ± SD or median and interquartile range as specified in the results. *p* < 0.05 were considered statistically significant.

## 3 Results

The characteristics of the study population are shown in Table 1. A total of 10 patients dropped out of the study, 5 patients during supine‐avoidance and 5 during CPAP, and one further patient had missing snoring data due to technical failure (faulty microphone) so was excluded from further analysis. Of the 66 participants in the SUPA‐OSA trial, 56 completed both treatment arms, and 15 (26.7%) met the predefined supine‐predominant snoring criteria of at least double the snoring frequency in supine compared to non‐supine sleep. The study sample was comprised of predominantly middle‐aged overweight men, as is typical in an OSA clinic sample and, by study selection criteria, exhibited at least mild through to severe supine‐predominant OSA.

**TABLE 1:** Baseline characteristics of the study population.

Parameter	Whole group	Supine snorer	Non‐supine snorer
*N* total	64	15 (23.4%)	49 (76.5%)
*N* females:males (%)	23:41 (36:64%)	4:11 (27:73%)	19:30 (39:61%)
Age (year)	52.8 ± 11.9	52.7 ± 10.9	54.6 ± 12.3
Height (cm)	171.9 ± 9.1	172.1 ± 9.2	171.9 ± 9.2
Weight (kg)	93.8 ± 21.4	87.3 ± 17.1	95.8 ± 22.3
Body mass index (kg/m^2^)	31.9 ± 7.8	29.7 ± 7.3	32.5 ± 7.9
Neck circumference (cm)	39.8 ± 3.8	39.0 ± 3.9	39.9 ± 3.8
Supine sleep time (%)	33.0 ± 24.6	45.4 ± 22.0	25.9 ± 22.0
Total AHI (/h)	14.2 ± 13.2	10.9 ± 10.5	13.4 ± 11.1
Supine AHI (/h)	25.4 ± 24.4	19.1 ± 14.8	24.5 ± 24.9
ESS	10.3 ± 3.9	9.9 ± 2.9	10.5 ± 4.2
AQoL—8D	69.6 ± 13.6	74.1 ± 10.2	68.3 ± 14.3
SSS	6.1 ± 2.6	6.4 ± 2.6	6.0 ± 2.6
Systolic blood pressure (mmHg)	127.8 ± 12.9	128.4 ± 14.6	127.2 ± 13.2
Diastolic blood pressure (mmHg)	79.0 ± 9.5	81.1 ± 8.0	78.1 ± 9.9
Total snoring frequency (snores ≥ 50 dBA/h)	48.9 [16.7 to 188.7]	26.3 [17.0 to 106.3]	57.5 [16.8 to 190.7]

*Note*: Values are mean ± SD or median [interquartile range].

Abbreviations: AHI, apnea hypopnea index; AQoL‐8D, assessment of quality of life; ESS, Epworth Sleepiness Scale; SSS, Snore Severity Scale.

During PSG at baseline, patients slept on their back for almost 30% of sleep and showed supine‐predominant OSA consistent with the study entry criteria applied to the pre‐study diagnostic PSG. There were no significant differences in any baseline characteristic between the supine‐predominant snorer sub‐group compared to the remaining group.

Table 2 shows snoring frequency in the full study sample at baseline and during repeat PSGs in the last week of each treatment. Compared to baseline, supine sleep time was significantly reduced with supine‐avoidance treatment (*p* < 0.001) but remained unchanged with CPAP. There were significant treatment by posture (*p* < 0.001), posture by snoring intensity (*p* = 0.017) and treatment by snore category (*p* < 0.001) effects on snoring frequency. Over the whole night, there was a reduction in snoring frequency with CPAP (*p* < 0.001) but not supine‐avoidance treatment (*p* = 0.651). CPAP also significantly reduced snoring frequency and intensity compared to baseline and supine‐avoidance therapy, in both supine and non‐supine sleep (percentage changes from baseline are reported in Table S1).

**TABLE 2:** Snoring frequency (snores/hour) of snoring by treatment group and snoring intensity separated by sleep posture and whole group.

Treatment	Baseline (*N* = 56)	Supine‐avoidance (*N* = 56)	CPAP (*N* = 56)
Supine sleep time (%)	30.7 [15.4 to 46.4]	0.2 [0.0 to 15.7]^a^, ^b^	36.7 [12.2 to 70.2]
AQoL—8D	69.5 [64.4 to 77.8]	71.3 [61.2 to 81.6]	73.4 [64.7 to 81.0]
SSS	6.0 [3.5 to 8.0]	5.5 [3.8 to 7.0]	6.0 [4.0 to 8.0]
Soft (≥ 50, < 60 dBA)	27.0 [7.7 to 87.8]	14.7 [3.8 to 99.2]	1.9 [0.7 to 5.7]^a^
Medium (≥ 60, < 70 dBA)	6.1 [1.4 to 40.9]	4.4 [0.3 to 55.4]	0.6 [0.3 to 2.6]^a^
Loud (≥ 70 dBA)	4.5 [1.6 to 26.0]	4.6 [0.8 to 42.5]	1.2 [0.5 to 6.2]^a^
Total (≥ 50 dBA)	48.9 [16.7 to 188.7]	36.8 [6.3 to 233.7]	4.2 [2.1 to 29.5]^a^, ^b^
During supine sleep			
Soft (≥ 50, < 60 dBA)	38.1 [10.9 to 79.2]	2.2 [0.0 to 19.1]^a^	3.5 [0.9 to 8.5]^a^
Medium (≥ 60, < 70 dBA)	9.2 [3.4 to 33.0]	0.0 [0.0 to 13.6]	1.0 [0.0 to 3.3]^a^
Loud (≥ 70 dBA)	5.3 [1.8 to 30.8]	1.3 [0.0 to 16.1]^a^	1.1 [0.0 to 12.1]^a^
Total (≥ 50 dBA)	66.3 [22.9 to 186.7]	13.2 [0.7 to 67.1]^a^	8.5 [1.6 to 44.3]^a^
During non‐supine sleep			
Soft (≥ 50, < 60 dBA)	19.8 [2.3 to 71.3]	13.7 [1.1 to 100.3]	1.5 [0.6 to 4.5]^a^
Medium (≥ 60, < 70 dBA)	3.4 [0.6 to 31.1]	2.6 [0.3 to 54.0]	0.5 [0.0 to 1.4]^a^
Loud (≥ 70 dBA)	3.4 [0.8 to 29.8]	2.4 [0.7 to 42.5]^a^	1.2 [0.5 to 4.8]^a^
Total (≥ 50 dBA)	33.8 [8.7 to 190.9]	36.8 [4.6 to 231.9]	4.0 [1.8 to 14.7]^a^, ^b^

*Note*: Values are median [interquartile range], *N* = 56.

Abbreviations: AQoL‐8D, assessment of quality of life; SSS, Snore Severity Scale.

^a^ *p* < 0.05 versus baseline.

^b^ *p* < 0.05 versus CPAP.

**FIGURE 1:**
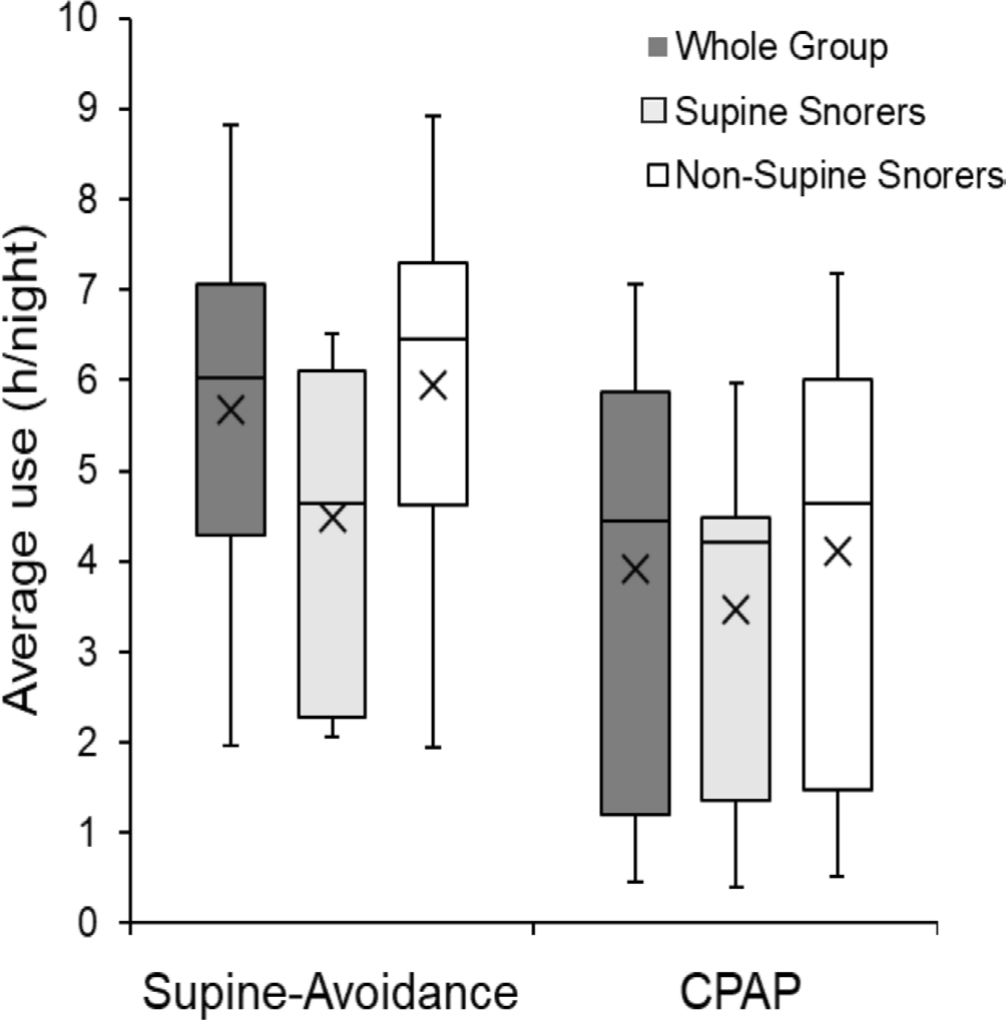
Average hours of use per night. Median and inter‐quartile ranges, 10%–90% whiskers, X indicates group mean over 6–8 weeks of treatment with supine‐avoidance compared to CPAP in the whole group (*N* = 56) and in supine‐predominant snorers (*N* = 15) compared to the remainder (*N* = 41) of the group.

Table 3 shows snoring frequency findings in the sub‐group of participants with supine‐predominant snoring expected to benefit most from supine‐avoidance therapy. As in the full study sample, this sub‐group showed a reduction in supine time with supine‐avoidance but not CPAP treatment. However, in contrast to the full group, these participants showed a significant reduction in total snoring frequency (treatment effect *p* < 0.001) and a shift to less severe snoring with both CPAP and supine‐avoidance therapy (posture effect *p* < 0.001, snoring intensity *p* < 0.001). However, snoring reductions were larger and more consistent with CPAP compared to supine‐avoidance, which, unlike CPAP (*p* = 0.026), did not significantly reduce soft and medium snoring (percentage changes from baseline are reported in Table S2). In addition, compared to baseline, non‐supine snoring was significantly reduced with CPAP (*p* < 0.001) but not supine‐avoidance (*p* = 0.299). Treatment type had no significant effect on AQoL or SSS scores in either the whole group (*p* = 0.294 and 0.327, respectively) or the supine snorer sub‐group (*p* = 0.345 and *p* = 0.896, respectively). Patients used the supine avoidance device more on an hourly basis every night compared to CPAP, but there were no differences between snoring phenotypes in device usage (Figure 1).

**TABLE 3:** Snoring frequency at baseline and on supine‐avoidance compared to CPAP treatment in patients with both supine‐predominant OSA and supine‐predominant snoring.

Treatment	Baseline (*N* = 15)	Supine‐avoidance (*N* = 15)	CPAP (*N* = 15)
Supine sleep time (%)	42.0 [35.3 to 54.3]	12.2 [0.0 to 22.6]^a^ ^,^ ^b^	32.6 [27.5 to 40.6]
AQoL—8D	75.2 [68.4 to 81.6]	70.2 [65.2 to 85.8]	78.0 [64.2 to 82.3]
SSS	6.5 [4.0 to 7.8]	6.0 [5.0 to 7.0]	7.0 [4.0 to 7.5]
Soft (≥ 50, < 60 dBA)	22.9 [12.8 to 71.6]	11.4 [2.1 to 15.0]	1.4 [0.7 to 3.7]^a^
Medium (≥ 60, < 70 dBA)	4.5 [2.4 to 15.9]	0.9 [0.2 to 2.6]	0.3 [0.2 to 0.9]^a^
Loud (≥ 70 dBA)	2.7 [1.5 to 4.6]	0.9 [0.3 to 3.0]	2.4 [0.7 to 3.7]
Total (≥ 50 dBA)	26.3 [17.0 to 106.3]	14.3 [3.0 to 18.1]^a^	4.6 [2.6 to 8.9]^a^
During supine sleep			
Soft (≥ 50, < 60 dBA)	48.7 [19.5 to 139.2]	7.8 [0.7 to 13.3]^a^	4.8 [1.8 to 5.5]^a^
Medium (≥ 60, < 70 dBA)	8.5 [5.2 to 43.9]	1.5 [0.0 to 3.0]^a^	0.9 [0.4 to 1.1]^a^
Loud (≥ 70 dBA)	3.9 [1.8 to 7.8]	2.1 [0.3 to 13.4]^a^	1.8 [0.4 to 3.5]^a^
Total (≥ 50 dBA)	57.8 [33.0 to 202.5]	11.1 [1.6 to 29.4]^a^	9.8 [4.4 to 10.2]^a^
During non‐supine sleep			
Soft (≥ 50, < 60 dBA)	4.2 [2.0 to 11.5]	5.9 [0.6 to 11.6]	0.7 [0.4 to 1.2]
Medium (≥ 60, < 70 dBA)	0.9 [0.3 to 1.7]	0.5 [0.2 to 1.4]	0.3 [0.2 to 0.4]
Loud (≥ 70 dBA)	0.9 [0.3 to 3.0]	0.9 [0.3 to 1.3]^a^	2.9 [0.8 to 5.1]
Total (≥ 50 dBA)	8.3 [3.3 to 17.1]	11.4 [3.3 to 17.1]	4.3 [2.4 to 5.7]^a^

*Note*: Values are mean [interquartile range], *N* = 56.

^a^ indicates *p* < 0.05 vs baseline.

^b^ *p* < 0.05 vs CPAP. Snore severity scale (SSS), Assessment of Quality of Life (AQoL‐8D).

## 4 Discussion

This is the first study to assess supine‐avoidance compared to CPAP treatment effects on objectively recorded snoring frequency and intensity in supine‐predominant OSA patients, and in a sub‐group of participants who also exhibited supine‐predominant snoring. These are amongst the most relevant patient groups for targeting supine‐avoidance device treatments. This is also one of very few studies to have examined the effects of CPAP on snoring, one of the most common symptoms and complaints for which patients seek sleep physician assessment and treatment (Krieger 1992; Guzman et al. 2017).

By study design patients were selected on the basis of supine‐predominant OSA, but with a normal non‐supine AHI; the OSA patient group most likely to benefit from supine‐avoidance treatment. In this group, successful supine‐avoidance should theoretically normalise the AHI and OSA specific symptomatology. However, although snoring and OSA are clear signs of a collapsible airway in sleep (Gleadhill et al. 1991), the frequency of transient partial or complete airway obstruction events, measured on the basis of AHI, may show little if any relationship with the frequency and intensity of snoring. Thus, problematic snoring could potentially occur throughout much of the sleep period despite a normal AHI and potentially despite treatments designed to improve airway function. In this study, only 23% of supine‐predominant OSA participants also exhibited supine‐predominant snoring, with markedly reduced (by a factor of at least 2) snoring in non‐supine sleep. Thus, successful supine‐avoidance in individuals with supine‐predominant OSA clearly does not necessarily translate into parallel improvements in snoring. This is an important finding and supports that more specific attention to snoring measurements and outcomes is clearly warranted in patients attending sleep clinic services, particularly when patients attend clinic on the basis of bed‐partner reported problem snoring. Nevertheless, snoring may be a strong sign of OSA, and given the importance of gravitational effects on the upper airway (Bilston and Gandevia 2014), supine‐avoidance is clearly worth considering as a treatment option in patients with supine‐predominant OSA or with a history of supine‐predominant snoring as the primary complaint.

By pneumatically splinting the upper airway to render partial and complete airway collapse less likely, CPAP might be expected to largely abolish snoring altogether. Whilst CPAP clearly did markedly reduce snoring in this patient group, some residual snoring remained, including loud snoring, particularly in supine sleep. Residual snoring potentially reflects periods of mask leak, and more challenging periods during the night such as supine and REM sleep where higher pressures could potentially achieve further reductions in snoring. CPAP was clearly superior compared to supine–avoidance in reducing snoring. However, in the sub‐group of patients with supine‐predominant snoring, supine‐avoidance was associated with a reduction in snoring comparable to CPAP. These findings support the need for careful patient selection and consideration of primary symptom complaints before selecting supine‐avoidance versus CPAP treatment. Of further note is the finding of considerably greater treatment adherence with supine‐avoidance compared to CPAP treatment, but not in the sub‐group of patients with supine‐predominant snoring. Whilst these findings are potentially confounded by multiple factors, including issues of statistical power given sub‐group analysis, these findings are strongly suggestive of important clinical trade‐offs between patient acceptance, comfort and perceived symptom relief.

There are no widely accepted standards for assessing and defining problematic snoring. Given that classic definitions of supine‐predominant OSA are based on at least a 2:1 ratio of supine versus non‐supine respiratory events (Cartwright et al. 1985), and perhaps clinically more usefully include a normal AHI in non‐supine sleep (Mador et al. 2005), we used a similar 2:1 ratio basis to define supine‐predominant snoring. This group could potentially benefit most from supine–avoidance, particularly when snoring is a primary complaint and given substantially poorly CPAP compared to supine‐avoidance use. However, what constitutes problematic snoring remains very poorly defined, and alternative definitions with a more specific focus around bed‐partner complaints and what might constitute acceptable residual snoring appear to be needed. Furthermore, in supine‐predominant snorers, treatment adherence was not different compared to CPAP, despite significant reductions in snoring, supporting the need for further studies to clarify what problems are most concerning to patients and/or their partners, balanced against treatment choices most likely to be efficacious and to be accepted and used by patients.

Given that this study analysed snoring in patients with supine‐predominant OSA, it is possible that outcomes may be different in participants with simple snoring alone without OSA. Alternative supine‐avoidance device approaches might also achieve different outcomes (Ha et al. 2014; Levendowski et al. 2015, 2014), although similar outcomes appear likely as long as the selected approach effectively achieves supine‐avoidance with minimal discomfort and sleep disruption (Rahimi, Vakulin, and Catcheside 2025). Nevertheless, improved outcomes might be possible through smarter devices able to detect and more specifically respond to posture‐dependent respiratory events or problem snoring without unnecessary alarm events during wake or quiet supine sleep without respiratory events that could potentially negatively impact patient acceptance and use.

In summary, this study showed that a simple vibration alarm‐based supine‐avoidance treatment in patients with supine‐predominant OSA does not necessarily translate into systematic reductions in snoring in sleep clinic patients with supine‐predominant OSA. Although CPAP clearly achieves superior OSA and snoring reduction outcomes, patient use over 6–8 weeks of treatment is substantially inferior, and some residual snoring remained even with CPAP. Yet in patients with both supine‐predominant OSA and supine‐predominant snoring, supine‐avoidance not unexpectedly achieves relatively effective treatment outcomes comparable to CPAP, including comparable treatment usage. Thus, there appear to be important and potentially quite complex trade‐offs between patient characteristics and treatment outcomes for which careful treatment selection is clearly warranted. These data support the principles of precision medicine and that in well‐selected patients, supine‐avoidance is a simple, viable and low‐cost approach well‐suited to patients with supine‐predominant OSA and/or snoring. Thus, comfortable non‐mask‐based treatments better targeted to specific abnormalities such as supine‐predominant snoring could help to manage a significant fraction of the community burden of sleep breathing disorders and help to achieve improved long‐term treatment outcomes, which are often poor.

## Author Contributions

**Matthew M. Rahimi:** writing – original draft, methodology, visualization, writing – review and editing, formal analysis, data curation. **Andrew Vakulin:** conceptualization, funding acquisition, supervision, writing – review and editing, methodology. **David Stevens:** writing – review and editing, formal analysis, methodology. **Peter G. Catcheside:** writing – review and editing, resources, supervision, conceptualization.

## Conflicts of Interest

The authors declare no conflicts of interest.

## Supporting information


**Data S1.** Supporting Information.

## Data Availability

The data that support the findings of this study are available from the corresponding author upon reasonable request.
