# Supplementary material for: A Comparison of Snoring Changes With a Supine‐Avoidance Alarm Device Compared to Constant Positive Airway Pressure Treatment in Patients With Supine‐Predominant OSA
Source: J Sleep Res. 2025 Jun 25;35(1):e70128. doi: 10.1111/jsr.70128 (PMC12856120; doi:10.1111/jsr.70128)
Supplement: Supplementary file 1 — Data S1. Supporting Information. [file JSR-35-e70128-s001.docx]

| **Treatment** | **Baseline (N=56)** | **Supine-avoidance (N=56)** | **% Change** | **CPAP (N=56)** | **% Change** |
| --- | --- | --- | --- | --- | --- |
| Supine Sleep time (%) | 30.7 [15.4 to 46.4] | 0.2 [0.0 to 15.7]*^ | - 99.3% | 36.7 [12.2 to 70.2] | + 19.4% |
| AQoL – 8D | 69.5 [64.4 to 77.8] | 71.3 [61.2 to 81.6] | + 2.6% | 73.4 [64.7 to 81.0] | + 5.6% |
| SSS | 6.0 [3.5 to 8.0] | 5.5 [3.8 to 7.0] | - 8.3% | 6.0 [4.0 to 8.0] | 0.0% |
| Soft (≥50, <60 dBA) | 27.0 [7.7 to 87.8] | 14.7 [3.8 to 99.2] | - 45.5% | 1.9 [0.7 to 5.7]* | - 92.9% |
| Medium (≥60, <70 dBA) | 6.1 [1.4 to 40.9] | 4.4 [0.3 to 55.4] | - 27.9% | 0.6 [0.3 to 2.6]* | - 90.2% |
| Loud (≥70 dBA) | 4.5 [1.6 to 26.0] | 4.6 [0.8 to 42.5] | + 2.2% | 1.2 [0.5 to 6.2]* | - 73.3% |
| Total (≥50 dBA) | 48.9 [16.7 to 188.7] | 36.8 [6.3 to 233.7] | - 24.8 % | 4.2 [2.1 to 29.5]*^ | - 91.4% |
| During Supine Sleep |  |  |  |  |  |
| Soft (≥50, <60 dBA) | 38.1 [10.9 to 79.2] | 2.2 [0.0 to 19.1]* | - 94.2% | 3.5 [0.9 to 8.5] * | - 90.8% |
| Medium (≥60, <70 dBA) | 9.2 [3.4 to 33.0] | 0.0 [0.0 to 13.6] | - 100% | 1.0 [0.0 to 3.3] * | - 89.1% |
| Loud (≥70 dBA) | 5.3 [1.8 to 30.8] | 1.3 [0.0 to 16.1]* | - 75.5% | 1.1 [0.0 to 12.1] * | - 79.2% |
| Total (≥50 dBA) | 66.3 [22.9 to 186.7] | 13.2 [0.7 to 67.1]* | - 80.0 % | 8.5 [1.6 to 44.3] * | - 87.1% |
| During Non-Supine Sleep |  |  |  |  |  |
| Soft (≥50, <60 dBA) | 19.8 [2.3 to 71.3] | 13.7 [1.1 to 100.3] | - 30.8% | 1.5 [0.6 to 4.5] * | - 92.4% |
| Medium (≥60, <70 dBA) | 3.4 [0.6 to 31.1] | 2.6 [0.3 to 54.0] | - 23.5% | 0.5 [0.0 to 1.4] * | - 85.3% |
| Loud (≥70 dBA) | 3.4 [0.8 to 29.8] | 2.4 [0.7 to 42.5]* | - 29.4% | 1.2 [0.5 to 4.8] * | - 64.7% |
| Total (≥50 dBA) | 33.8 [8.7 to 190.9] | 36.8 [4.6 to 231.9] | + 8.8% | 4.0 [1.8 to 14.7] *^ | - 88.2% |

**Table 1** Snoring frequency (snores/hour) of snoring by treatment group and snoring intensity separated by sleep posture and whole group. Values are median [interquartile range], N=56. * indicates p<0.05 vs baseline, ^ p<0.05 vs CPAP.Snore severity scale (SSS), Assessment of Quality of Life (AQoL-8D). % Change caluated from group level medians: ((Treatment − Baseline) / Baseline)×100

**Table 2.** Snoring frequency at baseline and on supine-avoidance compared to CPAP treatment in patients with both supine-predominant OSA and supine-predominant snoring.

| Treatment | Baseline (N=15) | Supine-avoidance (N=15) | % Change | CPAP (N=15) | %Change |
| --- | --- | --- | --- | --- | --- |
| Supine Sleep time (%) | 42.0 [35.3 to 54.3] | 12.2 [0.0 to 22.6]*^ | - 71.0% | 32.6 [27.5 to 40.6] | - 22.4% |
| AQoL – 8D | 75.2 [68.4 to 81.6] | 70.2 [65.2 to 85.8] | - 6.6% | 78.0 [64.2 to 82.3] | + 3.7% |
| SSS | 6.5 [4.0 to 7.8] | 6.0 [5.0 to 7.0] | - 7.7% | 7.0 [4.0 to 7.5] | + 7.7% |
| Soft (≥50, <60 dBA) | 22.9 [12.8 to 71.6] | 11.4 [2.1 to 15.0] | - 50.2% | 1.4 [0.7 to 3.7]* | - 93.9% |
| Medium (≥60, <70 dBA) | 4.5 [2.4 to 15.9] | 0.9 [0.2 to 2.6] | - 80.0% | 0.3 [0.2 to 0.9]* | - 93.3% |
| Loud (≥70 dBA) | 2.7 [1.5 to 4.6] | 0.9 [0.3 to 3.0] | - 66.6% | 2.4 [0.7 to 3.7] | - 11.1% |
| Total (≥50 dBA) | 26.3 [17.0 to 106.3] | 14.3 [3.0 to 18.1]* | - 45.6% | 4.6 [2.6 to 8.9]* | - 82.5% |
| During Supine Sleep |  |  |  |  |  |
| Soft (≥50, <60 dBA) | 48.7 [19.5 to 139.2] | 7.8 [0.7 to 13.3] * | - 84.0% | 4.8 [1.8 to 5.5] * | - 90.1% |
| Medium (≥60, <70 dBA) | 8.5 [5.2 to 43.9] | 1.5 [0.0 to 3.0] * | - 82.3% | 0.9 [0.4 to 1.1] * | - 89.4% |
| Loud (≥70 dBA) | 3.9 [1.8 to 7.8] | 2.1 [0.3 to 13.4] * | - 46.1% | 1.8 [0.4 to 3.5] * | - 53.8% |
| Total (≥50 dBA) | 57.8 [33.0 to 202.5] | 11.1 [1.6 to 29.4] * | - 80.8% | 9.8 [4.4 to 10.2] * | - 83.0% |
| During Non-Supine Sleep |  |  |  |  |  |
| Soft (≥50, <60 dBA) | 4.2 [2.0 to 11.5] | 5.9 [0.6 to 11.6] | + 40.4% | 0.7 [0.4 to 1.2] | - 83.3% |
| Medium (≥60, <70 dBA) | 0.9 [0.3 to 1.7] | 0.5 [0.2 to 1.4] | - 44.4% | 0.3 [0.2 to 0.4] | - 66.6% |
| Loud (≥70 dBA) | 0.9 [0.3 to 3.0] | 0.9 [0.3 to 1.3] * | 0.0% | 2.9 [0.8 to 5.1] | + 222.2% |
| Total (≥50 dBA) | 8.3 [3.3 to 17.1] | 11.4 [3.3 to 17.1] | + 37.3% | 4.3 [2.4 to 5.7] * | - 48.2% |
